# Evaluation of combined workflows for multimodal mass spectrometry imaging of elements and lipids from the same tissue section

**DOI:** 10.1007/s00216-024-05696-w

**Published:** 2025-01-20

**Authors:** Tassiani Sarretto, Mika T. Westerhausen, Jayden C. Mckinnon, David P. Bishop, Shane R. Ellis

**Affiliations:** 1 Molecular Horizons and School of Chemistry and Molecular Bioscience, University of Wollongong, Wollongong, Australia; 2 Hyphenated Mass Spectrometry Laboratory, University of Technology Sydney, Ultimo, Sydney, NSW Australia

**Keywords:** Mass spectrometry imaging, Multimodal imaging, Elemental analysis, Lipid analysis, Single tissue section

## Abstract

The wide range of mass spectrometry imaging (MSI) technologies enables the spatial distributions of many analyte classes to be investigated. However, as each approach is best suited to certain analytes, combinations of different MSI techniques are increasingly being explored to obtain more chemical information from a sample. In many cases, performing a sequential analysis of the same tissue section is ideal to enable a direct correlation of multimodal data. In this work, we explored different workflows that allow sequential lipid and elemental imaging on the same tissue section using atmospheric pressure laser desorption/ionisation-plasma post-ionisation-MSI (AP-MALDI-PPI-MSI) and laser ablation-inductively coupled plasma-MSI (LA-ICP-MSI), respectively. It is found that performing lipid imaging first using matrix-coated samples, followed by elemental imaging on matrix-coated samples, provides high-quality MSI datasets for both lipids and elements, with the resulting distributions being similar to those obtained when each is performed in isolation. The effect of matrix removal prior to elemental imaging, and of performing elemental imaging first were also investigated but found to generally yield lower quality elemental imaging data but comparable lipid imaging data. Finally, we used the ability to acquire both elemental and lipid imaging data from the same section to investigate the spatial correlations between different lipids (including ceramides, phosphatidylethanolamine, and hexosylceramides) and elements within mouse brain tissue.

**Graphical Abstract:**

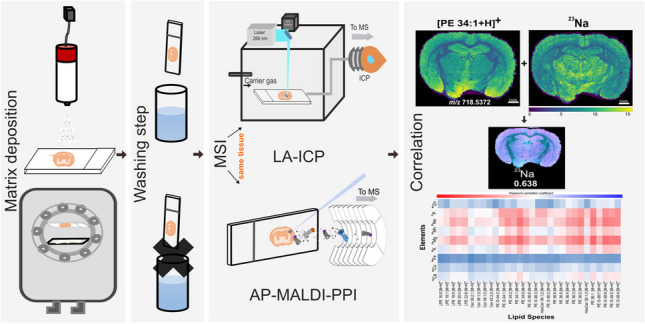

**Supplementary Information:**

The online version contains supplementary material available at 10.1007/s00216-024-05696-w.

## Introduction

Mass spectrometry imaging (MSI) has become a well-established technique to investigate a broad range of analytes such as glycans [1, 2], proteins [3–5], metabolites [6, 7], peptides [8], exogenous compounds [9–11], elements [12], and lipids [13, 14] in biological samples. The most widely used ionisation sources for imaging these analytes via MSI analysis include desorption electrospray ionisation (DESI) [15], secondary ionisation mass spectrometry (SIMS) [16], matrix-assisted laser desorption/ionisation (MALDI) [17, 18], and laser ablation-inductively coupled plasma (LA-ICP) [12]. Ionisation techniques can generally be classified as either soft, in that analytes are kept largely intact during desorption and ionisation, or hard whereby often extensive fragmentation/dissociation of analytes occurs during desorption and/or ionisation. MALDI, DESI, nano-DESI, and MALDESI represent examples of widely used soft desorption/ionisation techniques used for MSI and these are generally most suitable for MSI studies of biomolecules. Examples of hard desorption/ionisation techniques, include SIMS where often low mass fragments are observed, although it should be noted that the continued development of large cluster beams can greatly enhance the yield of intact ions compared to traditional atomic or small cluster sources [19], and LA-ICP-MS whereby analytes are ionised and atomised inside a plasma to yield elemental ions for analysis. LA-ICP-MSI is thus well suited for imaging of endogenous elements [20, 21] and metal-tagged antibodies [12, 22].

In recent years, the use of MSI has increased due to instrument advancements in sensitivity, speed, spatial resolution, and sampling capabilities. One area of rapid advance is the development of various post-ionisation techniques for MSI as a means to improve analyte coverage and sensitivity. These methods introduce a second ionisation event typically decoupled from the initial desorption/ionisation process and include laser post-ionisation [23–26], low-temperature plasmas [27–30], and photoionisation via the use of vacuum ultraviolet lamps [31, 32], often coupled with laser desorption or MALDI ion sources. For example, when applied to lipid imaging, post-ionisation techniques can significantly increase the sensitivity towards many lipid species, including those undetected in the absence of post-ionisation [33].

Given that MSI encompasses a broad range of ionisation techniques each with different analytical strengths and weaknesses, there is a significant interest in combining different MSI techniques on tissue sections to perform and combine MSI analysis of different analyte classes via a multimodal approach [34]. Multiplexed imaging workflows have been applied via both inter-MSI modalities, e.g. SIMS and MALDI [35–37], and also at targets not accessible via MSI, such as integration with spatial transcriptomics [38]. An example of this dual modality has been applied for the exploitation of immunohistochemistry with MALDI-MSI to perform label-free of hundreds of untargeted small biomolecules and targeted biomarkers on the same tissue section [39]. The approach incorporated photocleavable peptide tags for antibody labelling to enable multiplexed immunohistochemistry to be performed following lipid imaging using MALDI-MSI. Examples of multimodal applications of MALDI-MSI and LA-ICP-MS for elemental and biomolecular imaging have been used to investigate Zn distribution and its association with matrix metalloproteinase using sequential histological sections for each imaging modality [40], investigate image correlation between elements such as Au and Fe and selected lipid species from mice tissues sections injected with gold nanomaterial drugs [41], and investigate metal and lipid distributions within human multiple sclerosis tissue [42].

The spatial localisation of an analyte within a tissue supports unravelling its biological effects and functions, facilitating a more comprehensive understanding of cellular phenotypes and their structural organisation. Therefore, to enable correlation of multiple analyte classes that require different experiments to investigate it can be beneficial to perform analyses sequentially on the same tissue section to avoid any potential differences that can arise when using different sections which are often at least several cell layers apart. Feasibility of this multimodal imaging approach using the same tissue section has been employed via MALDI-MSI and LA-ICP-MSI to qualitatively and quantitatively investigate the distribution of arsenic-containing hydrocarbons in *Drosophila melanogaster* larvae [43]. Another example reported for molecular and elemental analysis on a single section was investigated by Holzlechner et al. that described a method to study the correlation between platinum, phosphorus, and lipid distributions on a malignant pleural mesothelioma sample tissue treated with cisplatin [44]. The workflow involved first subliming on a matrix for the MALDI-MSI approach, followed by the application of a thin layer of gold prior to LA-ICP-MS. These examples only analysed exogenous elements that are tightly bound in their chemical structure, and therefore behave differently to endogenous elements.

Sample handling is well known to negatively impact the concentrations and locations of endogenous metals; for example, sucrose protection on brain tissue resulted in redistribution of Zn in the hippocampus [45], and immunolabelling of muscle tissue caused significant changes in the concentration and redistribution of Zn and Cu [46]. With the potential of multimodal MSI to increase our knowledge of interactions between different classes of biologically active compounds and elements, it is necessary to determine the impacts of each imaging modality on the target analytes.

In this work, we have evaluated different experimental workflows for the sequential imaging of lipids and elements from the same tissue section at an atmospheric-pressure matrix-assisted laser desorption/ionisation platform with plasma post-ionisation (AP-MALDI-PPI) and LA-ICP-MS, respectively. Whilst analysis on elemental traces along with lipid distribution were previously assessed by LA-ICP-MSI and MALDI-MSI [40, 42–44], these methods often only focused on exogenous elements and/or performed analyses on separate tissue sections. Moreover, no systematic method comparison and evaluation of effects of matrix coating or the impact of order of acquisition between modalities for imaging analysis has been performed. Parameters investigated in this study include the effect of MALDI matrix and application method on LA-ICP-MS data, the suitability of matrix removal prior to LA-ICP-MS, and the order of acquisition [27]. Moreover, the work here utilises AP-MALDI-PPI, which offers complementary capabilities for lipid imaging compared to conventional MALDI. Exploiting the ability for multimodal analysis on the same section using optimised workflows where co-registration is straightforward, we also investigate the spatial correlations between different elements and lipids. This work provides insights into combinatorial workflows that enable lipid and elemental imaging from the same tissue section, expanding the repertoire of multimodal MSI techniques.

## Experimental methods

### Materials

Methanol (LC–MS grade), acetone (HPLC plus grade), xylene, and quick-hardening mounting medium were purchased from Sigma (Darmstadt, Germany). Water (LC–MS grade) was purchased from Merck (Darmstadt, Germany). Haematoxylin and eosin were purchased from POCD Scientific (North Rocks, NSW, Australia). Ethanol 100% undenatured was purchased from ChemSupply (Gillman, SA, Australia).

Elemental standards (1000 μg.mL^−1^) and Seastar Baseline nitric acid (HNO_3_) used for calibration and cross-quantification were supplied by Choice Analytical (Thornleigh, New South Wales, Australia). Tris–HCl (pH 7.4), ethylenediaminetetraacetic acid (EDTA), and gelatine from porcine skin (300 Bloom, Type A) were purchased from Sigma-Aldrich (Castle Hill, NSW, Australia).

### Sample preparation

Mouse brain tissue (8-month-old male) was provided as residual tissue from other animal studies at the University of Wollongong in accordance with the Australian code for the care and use of animals for scientific purposes. The mouse was euthanised by isoflurane, and the brain was flash-frozen immediately after collection using liquid nitrogen and maintained in storage under a controlled temperature at − 80 °C until sectioning.

**Fig. 1:**
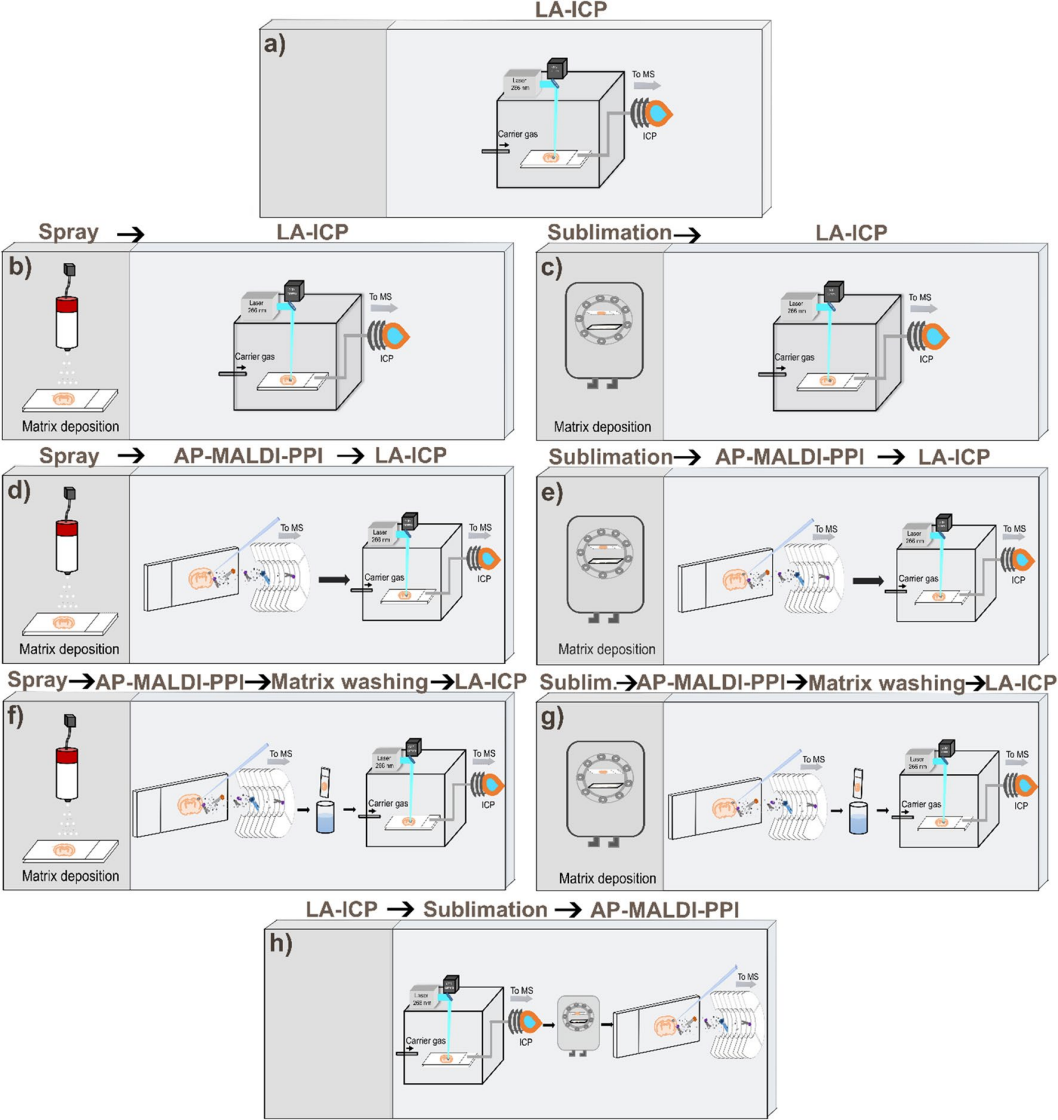
Slide protocols of sample preparation and mass spectrometry analysis. Slide A, analysis via LA-ICP without matrix. Slide B, LA-ICP analysis of spray-coated tissue section. Slide C, LA-ICP analysis of sublimation coated tissue section. Slide D, matrix coating via automatic sprayer followed by AP-MALDI-PPI analysis and LA-ICP. Slide E, matrix coating by sublimation followed by MS analysis via AP-MALDI-PPI and then LA-ICP. Slide F, matrix coating by automatic sprayer followed by AP-MALDI-PPI analysis, matrix washing, and LA-ICP analysis. Slide G, matrix coating via sublimation followed by AP-MALDI-PPI analysis, matrix washing and LA-ICP analysis. Slide H, analysis via LA-ICP followed by matrix coating using sublimation and AP-MALDI-PPI analysis

Consecutive coronal mouse brain sections with a 15-µm thickness were prepared using a cryo-microtome (Leica, Nussloch GmbH, Germany) at − 20 °C, thaw-mounted onto coated glass slides, and stored at − 80 °C until analysis. Prior to analysis, the tissue samples were retrieved from the freezer, placed into a vacuum desiccator, and allowed to reach room temperature over a time period of 30 min. Tissues were transported from the University of Wollongong to the University of Technology Sydney sealed with nitrogen at room temperature. Eight consecutive slides were analysed, each with differing sample preparation protocols (see Fig. 1).

### Matrix coating

Deposition of matrix via spray was performed using an automatic sprayer (TM-Sprayer, HTX Technologies, LLC, Chapel Hill, NC, USA). The matrix consisted of 2,5-dihydroxyacetophenone (DHA) dissolved in a solution of 70% ethanol to a concentration of 5 mg/mL and was applied to mouse brain sections using a flow rate of 0.03 mL/min for 20 layers, 10 psi of N_2_ pressure, 80 °C nozzle temperature, and a 1200 mm/min velocity. Deposition of the matrix via sublimation was performed using a home-built sublimation chamber. Sublimation was performed at 0.14 mbar using 20 mg of 2,5-DHA heated to 140 °C for 6 min.

### Tissue staining and microscopy

Tissue staining was performed using haematoxylin and eosin (H&E) on tissue sections following completion of MSI analyses. The matrix was first removed from the tissue by rinsing in methanol then were immersed in 95% ethanol, 70% ethanol, and water for 2 min each; then, they were immersed in haematoxylin and water for 3 min followed by immersion in eosin for 30 s and water for 3 min, and finally, immersion in 100% ethanol for 1 min and xylene for 30 s. The tissues were then air-dried and mounted with coverslips. For H&E-stained slides, optical images were acquired using a Leica SP8 FALCON (Leica Microsystems CMS GmbH, Mannheim, Germany) with a 10× objective.

### Instrumentation for AP-MALDI-PPI-MSI

Mass spectrometry imaging by AP-MALDI-PPI was performed using a timsTOF Pro mass spectrometer (Bruker Daltonics, Bremen, Germany) [47]. The instrument was coupled to a custom-built AP-MALDI source equipped with Smartbeam 3D 355 nm laser and a plasma post-ionisation source (Plasmion GmbH, Augsburg, Germany). A complete overview of the instrument was previously described [27]. The instrument was controlled using timsControl (Client version 3.0.0) and fleximaging (version 7.0, Bruker Daltonics GmbH & Co. KG). Mass calibration was performed using known endogenous lipid signals (list of peaks used for recalibration is provided in Electronic Supplementary Material Table S1). Mass spectra were acquired in the mass range of *m/z* 200–1000 in positive ion mode at a pixel size of 30 µm and the centre of the inlet capillary heated to 370 °C. One hundred laser shots were accumulated at each position using beam scanning dimensions of 20 × 20 µm. These laser parameters were selected to not fully ablate the matrix at each sampling position and thus leave the underlying tissue unaffected by the MALDI laser. Data was visualised using SCiLS Lab (version 2022b Pro, Bruker Daltonics GmbH & Co. KG) with a 10 ppm tolerance, total ion current normalisation, and 99% quantile hot-spot removal. A list of sum-composition identified lipids was manually created based on accurate mass using a 5 ppm m*/z* tolerance and matched to the LIPID MAPS database.

### Instrumentation for LA-ICP-MSI

An Elemental Scientific Lasers imageBIO266 laser ablation system (Kenelec Scientific, Mitcham, Victoria, Australia) coupled to an Agilent Technologies 7900 Series ICP-MS (Agilent Technologies, Mulgrave, Victoria, Australia) was used for all experiments. Instrument parameters are shown in Table 1. For the reduced laser energy experiment, the laser energy was dropped until two passes were required to fully ablate the tissue within a line scan.

**Table 1:** Instrument parameters for LA-ICP-MS

Laser ablation	Conditions
Wavelength	266 nm
Laser power	15 and 20%
Laser fluence	2800 and 3750 mJ.cm^−2^
Laser beam diameter	30 µm
Scan speed	750 µm/s
Frequency	200 Hz
ICP-MS	
RF power source	1350 W
Carrier gas (L min)	0.7 He
Makeup gas (mL min)	0.45 Ar
Sample depth	4 mm
Extract lens 1 and 2	4.5 V, − 125 V
Omega bias, lens	− 80 V, 13.2 V
Octopole RF	180 V
Octopole bias	− 18 V
Collision gas	3.1 mL min^−1^ H_2_

### External calibration standards

Experiments performed on standards (Mn, Fe, Cu, Zn) used for quantification were prepared according to a previously validated protocol [48]. Briefly, 100 mg of porcine gelatine powder was spiked with metal solutions and made up to 1 mL using a 100 mM tris and 10 mM EDTA buffer solution. The gelatine solution was vortexed and heated at 50 °C until dissolved and pipetted into pre-heated slides with HybriWell™ (Sigma-Aldrich) sealing systems attached. Slides were frozen at − 80 °C for 5 min, and the sealing system was removed and then dried in a desiccator overnight.

### Visualisation and correlation analysis

LA-ICP-MS samples were visually inspected and quantified using Pew2 [49] before being exported as CSV files. For isotopes not quantified by external calibration standards (^23^Na, ^44^Ca, ^31^P, and ^39^ K), the raw counts per second values are shown. The lack of quantification is due to the high background signal of these elements found endogenously in gelatine.

For correlation analysis, TIC-normalised MALDI images were extracted using Cardinal v3.0 [50] as CSV files. Two stacks of CSV files, one containing LA-ICP-MS images and one containing MALDI images, were imported into FIJI [51] and registered to one another using the Multi-Image Landmark Correspondences plugin by David Cohoe (https://github.com/sudgy/multi-landmark). The registered stacks were then compared pixel-to-pixel by Pearson’s correlation coefficients for correlation generated using an in-house written MATLAB code (Electronic Supplementary Material Code S1) [52]. False colour composite images of red and blue or magenta and cyan comparing the high and low correlation between LA-ICP-MS and AP-MALDI-PPI ions were generated using FIJI.

## Results and discussion

The purpose of this research was to evaluate different protocols for performing both elemental and lipid imaging on a single tissue section using LA-ICP-MSI and AP-MALDI-PPI-MSI. A variety of protocol combinations were evaluated on a single tissue section via different MSI modalities to explore the effects of matrix application, matrix washing, and the order of acquisition. These workflows are summarised in Fig. 1 and are discussed further below.

### Effects of 2,5-DHA matrix coating on elemental imaging using LA-ICP-MSI

Whilst a MALDI-like matrix is not necessarily needed for ionisation via AP-MALDI-PPI, the absence of a matrix would result in low desorption efficiency of biomolecules from the tissue section using a 355-nm laser. Thus, a UV absorbing matrix is used for AP-MALDI-PPI to allow for broad and soft desorption of lipids from the tissue section into the inlet capillary, where they are subsequently ionised by the cold plasma source. It also provides direct relevance to conventional MALDI-MSI workflows. In contrast, LA-ICP-MSI uses short wavelength UV lasers and higher pulse energies (266 nm used here) and combined with no requirements for enabling analyte desorption with minimal fragmentation, a MALDI-like matrix is not required for LA-ICP-MSI. Therefore, to first investigate the influence of matrix coating on elemental imaging, one mouse brain section was analysed by LA-ICP in the absence of matrix (Fig. 1a, reflective of a conventional LA-ICP-MSI experiment) and two brain sections coated in 2,5-DHA matrix, which was previously shown to perform well for lipid imaging using AP-MALDI-PPI [27, 30]. The tissue section in Fig. 1b was matrix coated via an automated sprayer and the tissue section in Fig. 1c was coated using sublimation. The investigation of both spraying and sublimation covers the two main application methodologies used for MALDI-MSI studies. To date, the effect of MALDI-like matrices on the imaging of metals in biological tissues has not been investigated in detail. However, assessing whether the matrix application influences elemental distribution in biological tissues is of utmost importance for protocol optimisation of multimodal analysis as the workflow can be simplified and time saved removing one extra step between MSI analysis. Moreover, assessing the advantages of imaging elements with the matrix still coated on the tissue might prevent the need for matrix washing as this process poses the risk of analyte delocalisation.

**Fig. 2:**
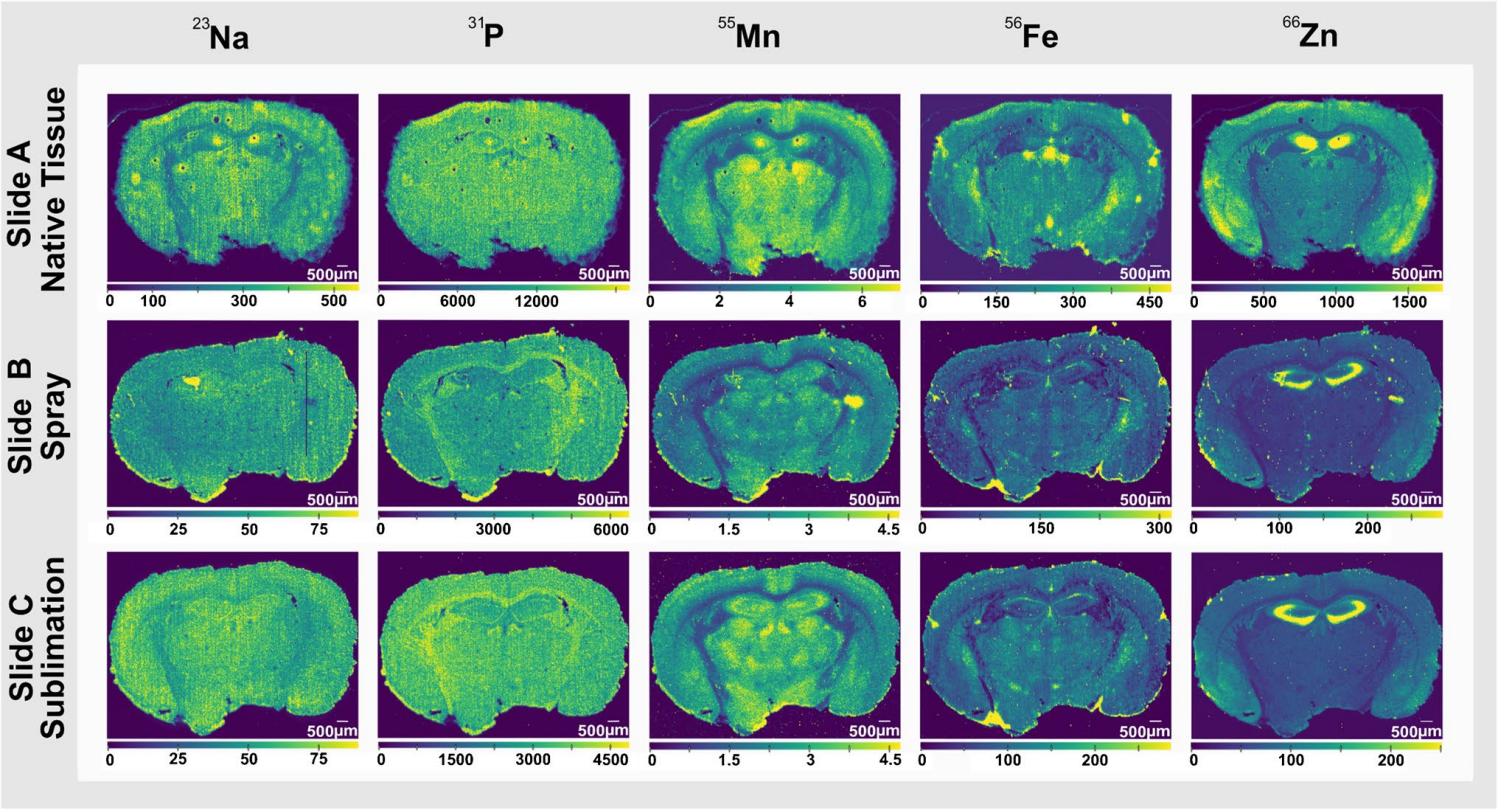
Elemental analysis acquired via LA-ICP-MSI of mouse brain tissue sections under different conditions. Slide **A** was analysed without matrix deposition. Slide **B** was analysed with matrix deposition via an automated sprayer. Slide **C** was analysed with matrix deposition via sublimation. ^55^Mn, ^56^Fe, and ^66^Zn are in mg/kg, and ^23^Na and ^31^P are in counts/second (see “Experimental methods”)

A broad range of elements were targeted for this study, including the distribution of ^23^Na, ^24^ Mg, ^31^P, ^39^ K, ^44^Ca, ^55^Mn, ^56^Fe, ^63^Cu, and ^66^Zn. Figure 2 shows the distribution of ^23^Na, ^31^P, ^55^Mn, ^56^Fe, and ^66^Zn across the native tissue (top row), spray-coated sample (middle row), and sublimation-coated sample (bottom row). The remaining elemental images for slides A, B, and C can be found in Electronic Supplementary Material Fig. S1.

The native tissue (slide A) in Fig. 2 shows a higher signal intensity for many elements than tissues coated with matrix (slides B and C), this being more visually pronounced for the distribution of ^31^P, ^39^ K, and ^66^Zn when quantifying using external calibration standards not coated with matrix. This could be rationalised due to the influence that the matrix plays on the optimum quantity of molecules/ions ablated for each scan, suppressing elemental yields. High concentrations of non-analyte isotopes have been shown to suppress analyte signals in ICP-MS due to changes in the plasma temperature, aerosol transport efficiency of the sample, and MS conditions [53, 54].

**Fig. 3:**
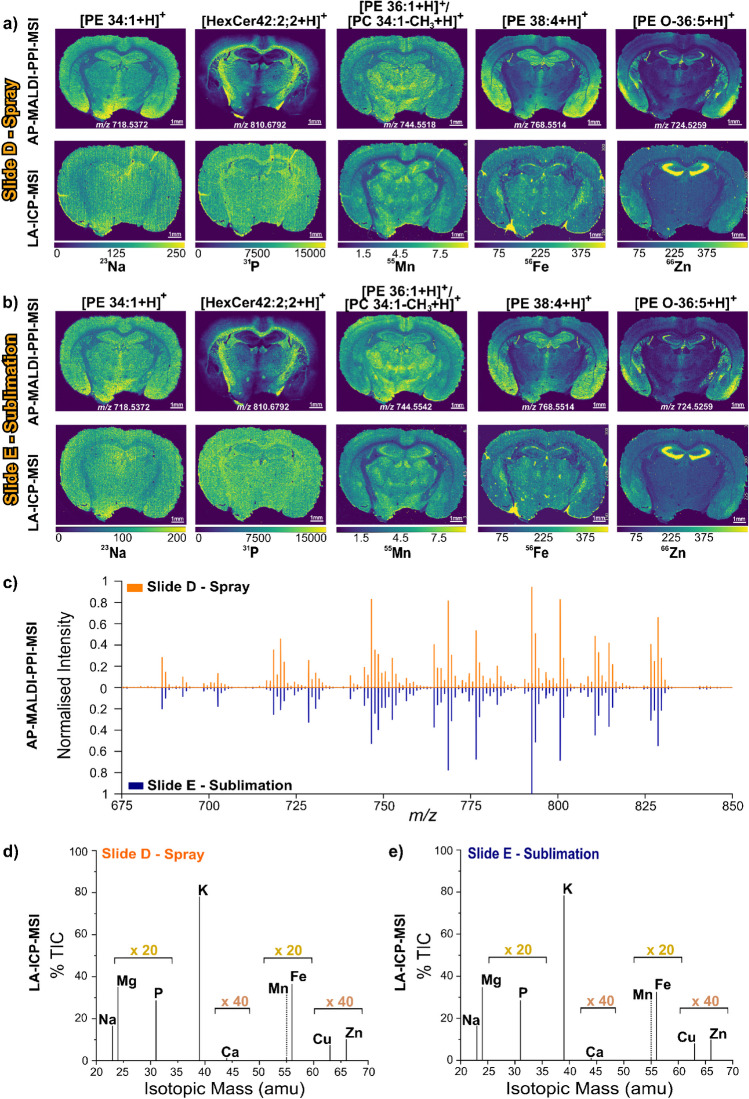
Molecular and elemental distributions of slides D and E. **a** Selected lipid (top row) and elemental (bottom row) distribution images of slide D that were analysed using spray-based matrix coating. **b** Selected lipids (top row) and elemental that were analysed using sublimation-based matrix coating. In both cases, AP-MALDI-PPI-MSI was performed first followed by LA-ICP-MSI. ^55^Mn, ^56^Fe, and ^66^Zn are in mg/kg and ^23^Na and ^31^P are in counts/second (see “Experimental methods”). **c** Butterfly plot of AP-MALDI-PPI averaged mass spectra for spray-coated (top, orange trace) and sublimation-coated (bottom, blue trace) tissue sections. **d** Averaged LA-ICP MS mass spectrum of spray-coated (left) and sublimation-coated (right) tissue sections. Only the elements labelled were quadrupole selected and detected during LA-ICP-MS imaging. 20 × and 40 × signify the magnification of the given elemental signal so it is visually perceivable in the plots for comparison

Elemental distributions throughout the mouse brain tissues in Fig. 2 yielded specific distributions for each element selected. Whilst ^23^Na and ^55^Mn appear with a similar localisation within the tissue, ^56^Fe and ^66^Zn exhibit a more distinct distribution. ^56^Fe is localised more on the third ventricle and caudoputamen, whilst ^66^Zn is found at higher concentrations in the CA1, CA2, and CA3 field of the hippocampus, molecular layer dentate gyrus along with more intense regions in the cortical subplate and olfactory areas. There are several hot-spots observed for ^56^Fe that colocalise between slides A–C as well as those shown in Fig. 3. The colocalisation suggests these are not technical artefacts. We hypothesise this may be due to residual blood or blood vessels present within the tissue. The ^31^P distribution shows a relatively homogeneous distribution for all three samples. The spatial distributions of elements herein outlined are consistent with previous reports [55–57].

The most prominent difference in elemental imaging between the three workflows is observed for ^66^Zn distribution, where the enriched area in the stratum radiatum and stratum lacunosum-moleculare region observed for slide A shifts to the outer pyramidal cell layer in the hippocampus in the matrix-coated samples (slides B and C, Fig. 1). One hypothesis for this shift is redistribution caused by matrix application. Extensive studies have shown the impact that sample preparation has had on the disruption and depletion of Zn distributions [58–61]. Another explanation for the different ^66^Zn distributions is due to the different coronal regions of the mouse brain, with the tissue section in slide A being approximately 100 µm away from slides B and C but closer in proximity from slides F and G, where the distribution of slide A seems to more closely resemble the distribution observed for slides F and G. This similarity with slides F and G suggests the distribution of ^66^Zn shown in slide A is reflective of the brain regions shown. With sections, optical images of H&E-stained tissues post acquisition are provided in Electronic Supplementary Material Fig. S5.

Despite the effect of the matrix on LA-ICP-MSI signal intensities, the relative elemental distributions observed for slides B and C remain comparable for most of the investigated elements, with Zn as a notable exception, that might be explained by factoring in possible differences due to the bregma coordinates of each section. No major differences in elemental distributions are observed when comparing different matrix applications [62]. These results demonstrate that tissue coated in MALDI matrices is still amenable to LA-ICP-MSI analysis, a critical observation if one is to perform biomolecular imaging using MSI prior to LA-ICP-MSI analysis.

### Evaluation of sequential lipid and elemental imaging using 2,5-DHA-coated tissues

Next, we investigated sequential lipid and elemental imaging on matrix-coated tissues using AP-MALDI-PPI-MSI followed by LA-ICP-MSI. Slides D and E (Fig. 1) were sprayed and sublimated-coated, respectively, and then analysed via AP-MALDI-PPI-MSI followed by LA-ICP-MSI.

Lipid distributions were investigated in positive ion mode via AP-MALDI-PPI, which resulted in the generation of predominantly protonated lipid signals. Consistent with earlier reports, the dominant signals following AP-MALDI-PPI of brain tissue were from hexosylceramides (HexCer) and phosphatidylethanolamine (PE) lipid species as well as demethylated phosphatidylcholine (PC) [27, 30]. A range of well-known lipid species classified as [PE 34:1 + H]^+^ at *m/z* 718.5372, [HexCer 42:2;2 + H]^+^ at *m/z* 810.6792, [PE 36:1 + H]^+^/[PC 34:1-CH_3_ + H]^+^ at *m/z* 744.5518, [PE 38:4 + H]^+^ at *m/z* 768.5514, and [PE O-36:5 + H]^+^ at *m/z* 724.5259 were selected for visualisation and are shown for slides D (spray coated) and E (coated via sublimation) in the top rows of Fig. 3a and b, respectively. The corresponding averaged mass spectra within the lipid mass range for the spray and sublimation-coated samples are shown in Fig. 3c.

Following AP-MALDI-PPI-MSI, elemental imaging via LA-ICP-MSI was performed on the matrix-coated tissues. The elemental images for ^23^Na, ^31^P, ^55^Mn, ^56^Fe, and ^66^Zn for spray and sublimation-coated slides are shown in the bottom rows of Fig. 3a and b, respectively. The corresponding averaged LA-ICP-MSI spectra are shown in Fig. 3d. Critically, elemental distributions for both coating methods following AP-MALDI-PPI-MSI are similar for slides A–C (Fig. 2), with the distribution of ^66^Zn also being similar to that measured for slides B and C shown in Fig. 2. The LA-ICP-MSI signal intensities were also similar between the two coating methods. Higher elemental signal intensities were observed for slides that underwent prior AP-MALDI-PPI-MSI analysis (Fig. 3a, b) compared to matrix-coated slides that did not (Fig. 2). This is believed to have taken place due to the desorption of the matrix layer performed under AP-MALDI-PPI-MSI analysis, which competes with the total amount of molecules/clusters ablated, consequently suppressing elemental yields. Critically, though, these data demonstrate the feasibility of performing sequential lipid and elemental MSI on the same tissue section, even when using a MALDI matrix to enable efficient desorption and ionisation of lipids.

### Effect of matrix washing on sequential lipid and elemental imaging

Whilst the above results show that LA-ICP-MSI can be performed on matrix-coated samples following biomolecular imaging, in some cases, it may be desirable to remove the matrix prior to analysis. Thus, we next explored the effects of adding a matrix-washing step between AP-MALDI-PPI-MSI and LA-ICP-MSI acquisition. The tissue sections were coated with 2,5-DHA, either by spray (slide F) or sublimation (slide G), analysed via AP-MALDI-PPI-MSI, matrix washed by rinsing the slide with methanol, and then analysed via LA-ICP-MSI.

**Fig. 4:**
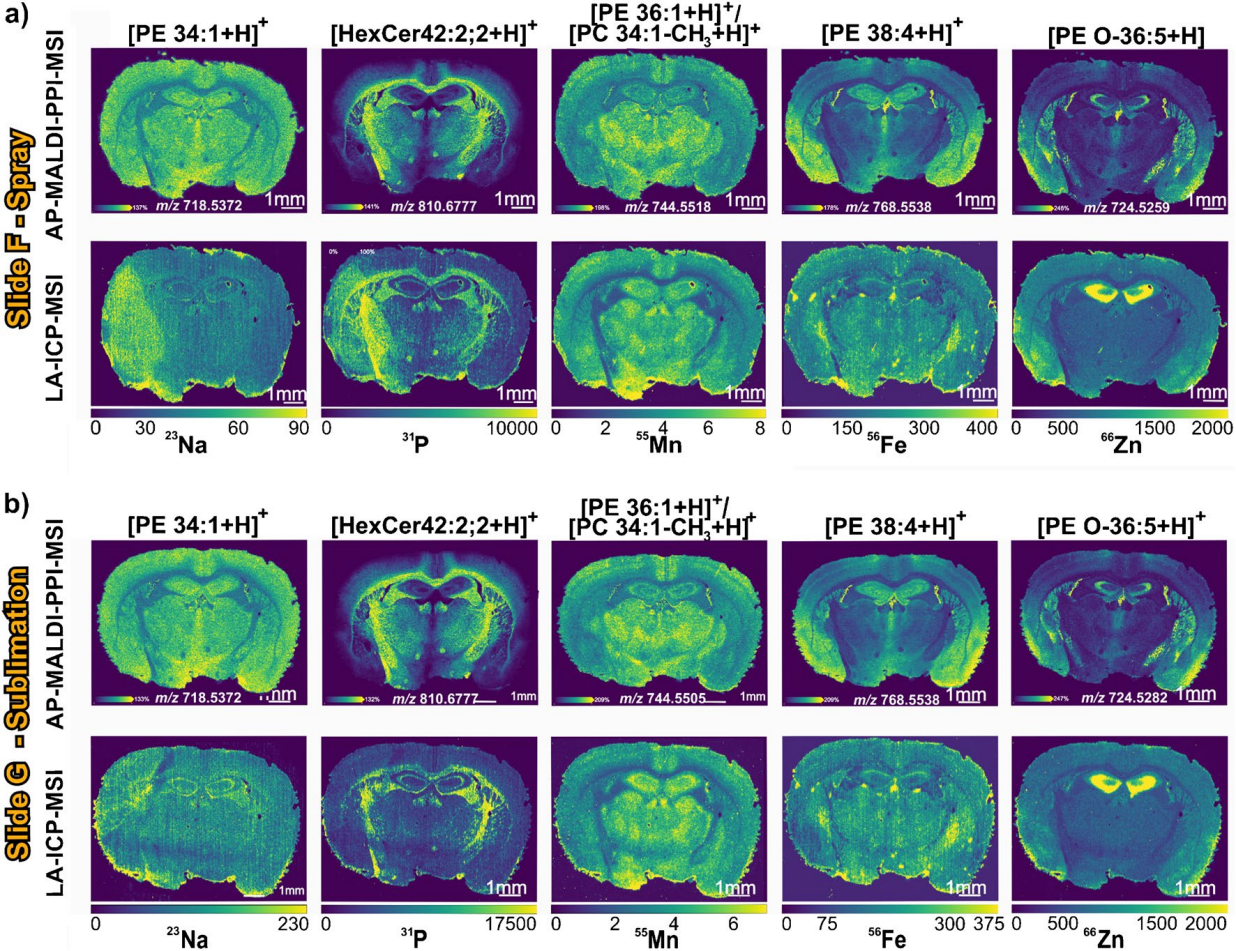
Molecular and elemental distributions of slides F and G. **a** Molecular and elemental distribution images of slide F that was coated using spray-based matrix deposition, analysed by AP-MALDI-PPI-MSI, matrix washed, and then analysed via LA-ICP-MSI. **b** Molecular and elemental distribution images of slide G that was matrix coated via sublimation, analysed via AP-MALDI-PPI-MSI, matrix washed, and then analysed via LA-ICP-MSI. Ion images are visualised with a 10 ppm tolerance, total ion current normalisation, and 99% quantile hot-spot removal. ^55^Mn, ^56^Fe, and ^66^Zn are in mg/kg and ^23^Na and.^31^P are in counts/second (see “Experimental methods”)

As expected, the overall lipid profile obtained using both matrix coating methods was similar (see spectra in Electronic Supplementary Material Fig. S3). The spatial distribution of several selected lipids, namely, [PE 34:1 + H]^+^, [HexCer 42:2;2 + H]^+^, [PE36:1 + H]^+^/[PC 34:1-CH_3_ + H]^+^, [PE 38:4 + H]^+^, and [PE O-36:5 + H]^+^ species also remains similar throughout the biological tissue for both approaches (Fig. 4a and b), and also for data shown in Fig. 3.

Interestingly, following matrix removal, the intensity and distribution images of ^55^Mn, ^56^Fe, and ^66^Zn for both datasets more closely resemble those obtained without matrix removal and from the native tissue sections shown in Figs. 2 and 3. These relatively water-insoluble elements are tightly bound into various proteins within the brain, the most relevant of which are: Mn is found in glutamine synthetase, an essential enzyme in astrocytes [63]; Fe is incorporated into neurons, microglia, and astrocytes via binding to transferrin [64]; and Zn is bound to metallothioneins with 3 of the 4 isoforms being expressed exclusively in the CNS, primarily in astrocytes [65].

In contrast, from the spray-coated slide, the spatial trace for ^23^Na, ^31^P (Fig. 4a), and ^39^ K (Electronic Supplementary Material Fig. S4) reveals distribution artefacts on the left side of the tissue on slide F. This likely arises due to the washing causing delocalisation of some elements, or biomolecules containing those elements. Element mobility after aqueous washing has been observed previously with lighter, water-soluble group I and II elements such as ^23^Na and ^39^ K either being leeched out of or redistributed within mouse brain samples [66]. The observation that some metals, like ^23^Na and ^39^ K, are delocalised upon solvent treatment whilst others such as ^55^Mn are not is also consistent with other studies investigating the influence of formalin fixation and paraffin embedding on elemental images obtained with LA-ICP-MS [67]. Interestingly, the sublimation-coated slide does not display the same elemental artefacts (Fig. 4b and S4), possibly suggesting that elemental delocalisation due to washing can be highly variable.

Despite delocalisation negatively affecting image quality, the ^23^Na and ^31^P signal intensities, after washing the matrix off for both coating modalities, remain relatively similar when compared to the protocol that does not include a washing step on slides D and E (Fig. 3), although it should be noted that delocalisation on scales smaller than the pixel used employed here could be present. In contrast, the lateral distribution images of ^66^Zn for slides F and G show an increase in intensity when adding a washing step compared to slides D and E. This increase in intensity for ^66^Zn distribution might have arisen due to the removal of the matrix that is believed to suppress and deplete the zinc signal as observed in Fig. 2, where the intensity for Zn was at its highest without matrix application. And the elemental distribution for ^55^Mn and ^56^Fe remains consistent for both protocols.

These results further demonstrate the possibilities for performing both lipid and elemental MSI sequentially on the same tissue section and that matrix washing prior to LA-ICP-MSI still preserves the distributions of many elements. However, analysis of matrix coated without washing appears to be most favourable for obtaining good-quality LA-ICP-MSI on the broadest range of elements. It should be noted that the exact nature of any delocalisation (e.g. the extent of delocalisation and the elements it impacts) will depend on the protocol deployed for washing.

### Performing LA-ICP-MSI prior to AP-MALDI-PPI-MSI

**Fig. 5:**
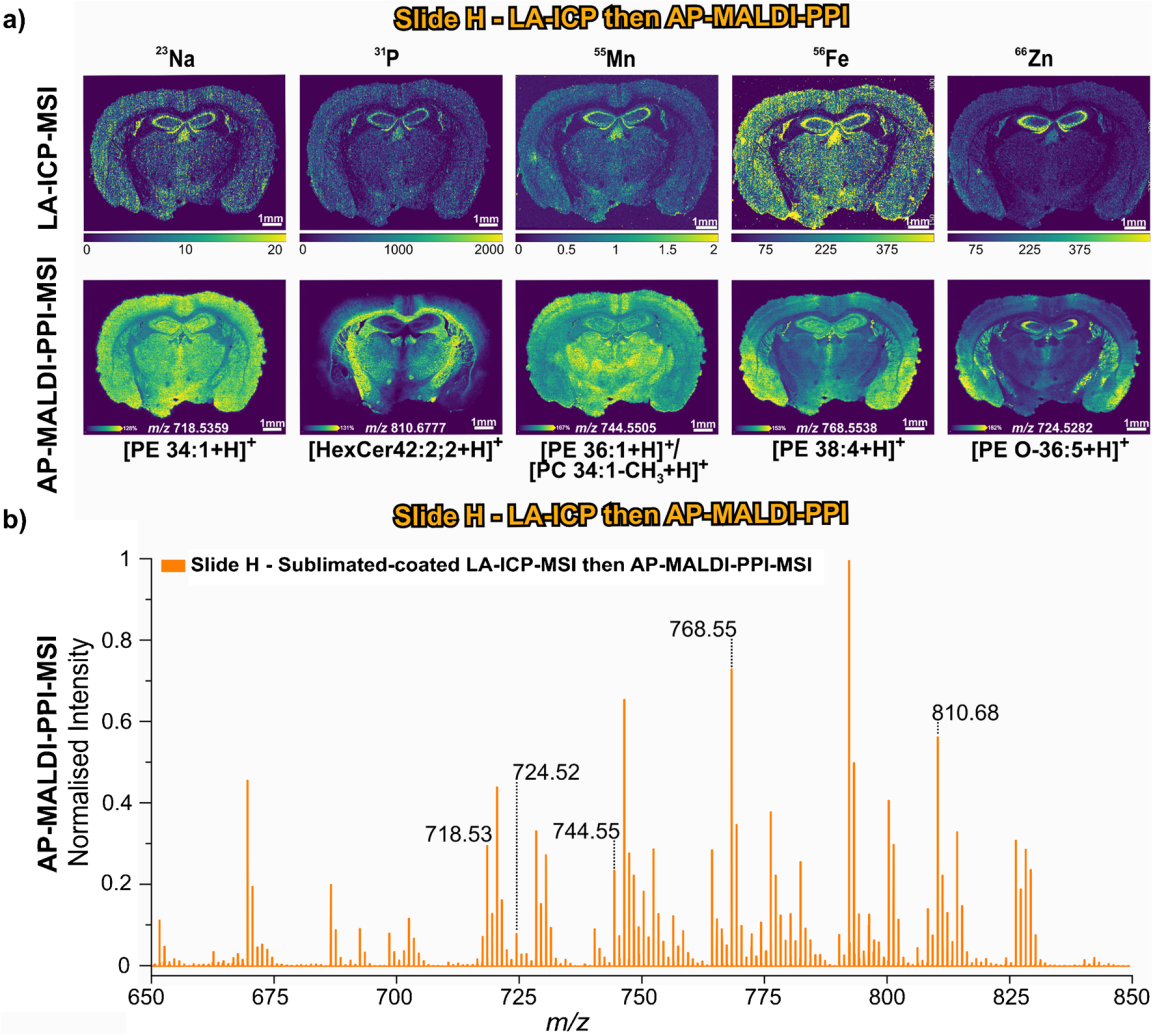
Spatial and spectral data of elemental and molecular MSI workflow outlined on slide H. **a** Elemental (top row) and molecular (bottom row) distribution images of slide H acquired via LA-ICP-MSI analysis then matrix coated using sublimation and analysed by AP-MALDI-PPI-MSI. **b** Averaged mass spectra for slide H outlining the five selected ions shown on **a**. ^55^Mn, ^56^Fe, and ^66^Zn are in mg/kg and ^23^Na and.^31^P are in counts/second (see “Experimental methods”)

Finally, we investigated the possibility of performing LA-ICP-MSI before lipid imaging by AP-MALDI-PPI-MSI. Standard LA-ICP-MSI conditions (i.e. those used to acquire data in Figs. 2, 3, and 4) completely ablated the tissue, as would be expected with the continuous raster approach and requirements for complete abaltion, and were therefore unsuitable for subsequent lipid imaging (refer to post-MSI stained tissue sections in Electronic Supplementary Material Fig. S5). Therefore, for this investigation, the laser power for LA-ICP-MSI was reduced to reduce the amount of tissue ablation. This was indeed confirmed by post-MSI staining and optical imaging (Electronic Supplementary Material Fig. S5). Following LA-ICP analysis, the tissue section was coated in 2,5-DHA via sublimation and then analysed by AP-MALDI-PPI-MSI. The results of this analysis are shown in Fig. 5. The top row of Fig. 5a shows the elemental images of ^23^Na, ^31^P, ^55^Mn, ^56^Fe, and ^66^Zn and the bottom row the ion distribution of [PE 34:1 + H]^+^, [HexCer 42:2;2 + H]^+^, [PE 36:1 + H]^+^/[PC 34:1-CH_3_ + H]^+^, [PE 38:4 + H]^+^, and [PE O-36:5 + H]^+^ species.

AP-MALDI-PPI-MSI following LA-ICP-MSI at reduced laser energy revealed similar ion images for lipids (Fig. 5a, top row) compared to the workflows discussed above where AP-MALDI-PPI-MSI was acquired prior to elemental analysis. Figure 5b shows the averaged mass spectra for the relevant lipid mass range for slide H (full mass range spectra are provided in Electronic Supplementary Material Fig. S6). Not only was the signal intensity similar to that acquired when lipid imaging was performed first but no notable evidence of significantly increased lipid fragmentation caused by irradiation with the LA-ICP laser was observed, thereby demonstrating the capabilities of profiling lipids on biological tissues post-laser ablation.

Although the lipid profiling acquired for this acquisition sequence was similar to earlier workflows, the elemental distribution in Fig. 5a shows notable differences in terms of both overall signal intensity and distribution. For example, distributions of ^55^Mn and ^66^Zn were overall similar to those shown in Fig. 2. However, for some, overall signal intensity and image contrast are reduced. The ion images of ^31^P and ^23^Na show a different preferential localisation. The distribution of ^31^P and ^23^ Na is relatively homogeneously distributed for the workflows shown in Figs. 2, 3, and 4. However, under lower laser energy conditions, both show a significant reduction in signal throughout the tissue, with signals preferentially localised more to the pyramidal cell layer in the hippocampus and in the third ventricle. Even though, the ^56^Fe distribution shows certain similarities, such as intensities being concentrated in the third ventricle for both workflows (Figs. 5 and 2), the high predominance of intensity in the hippocampus in Fig. 5 is not observed in the native tissue (Fig. 2). A possible rationalisation could be related to the only partial, and region-dependent, ablation efficiencies across the tissue under these reduced and non-ideal ablation laser energies (see post-MSI optical image of slide H in Electronic Supplementary Material Figure S5) [68]. Whilst this workflow demonstrates the feasibility of lipid imaging acquisition after LA-ICP, the above results support the idea that it is beneficial to perform lipid imaging prior to elemental imaging using LA-ICP.

### Correlation analysis of endogenous elements and lipids

The ability to acquire elemental and lipid distributions from the same tissue section led us to investigate the spatial correlations between different elements and selected lipid species. For this, we used the workflow outlined for slide E (Figs. 1 and 3) where the tissue section was coated with 2,5-DHA via sublimation and then analysed sequentially by AP-MALDI-PPI and LA-ICP. We note that this is in contrast to previous studies that have performed co-registration of MALDI and LA-ICP-MS data using adjacent tissue sections [41].

**Fig. 6:**
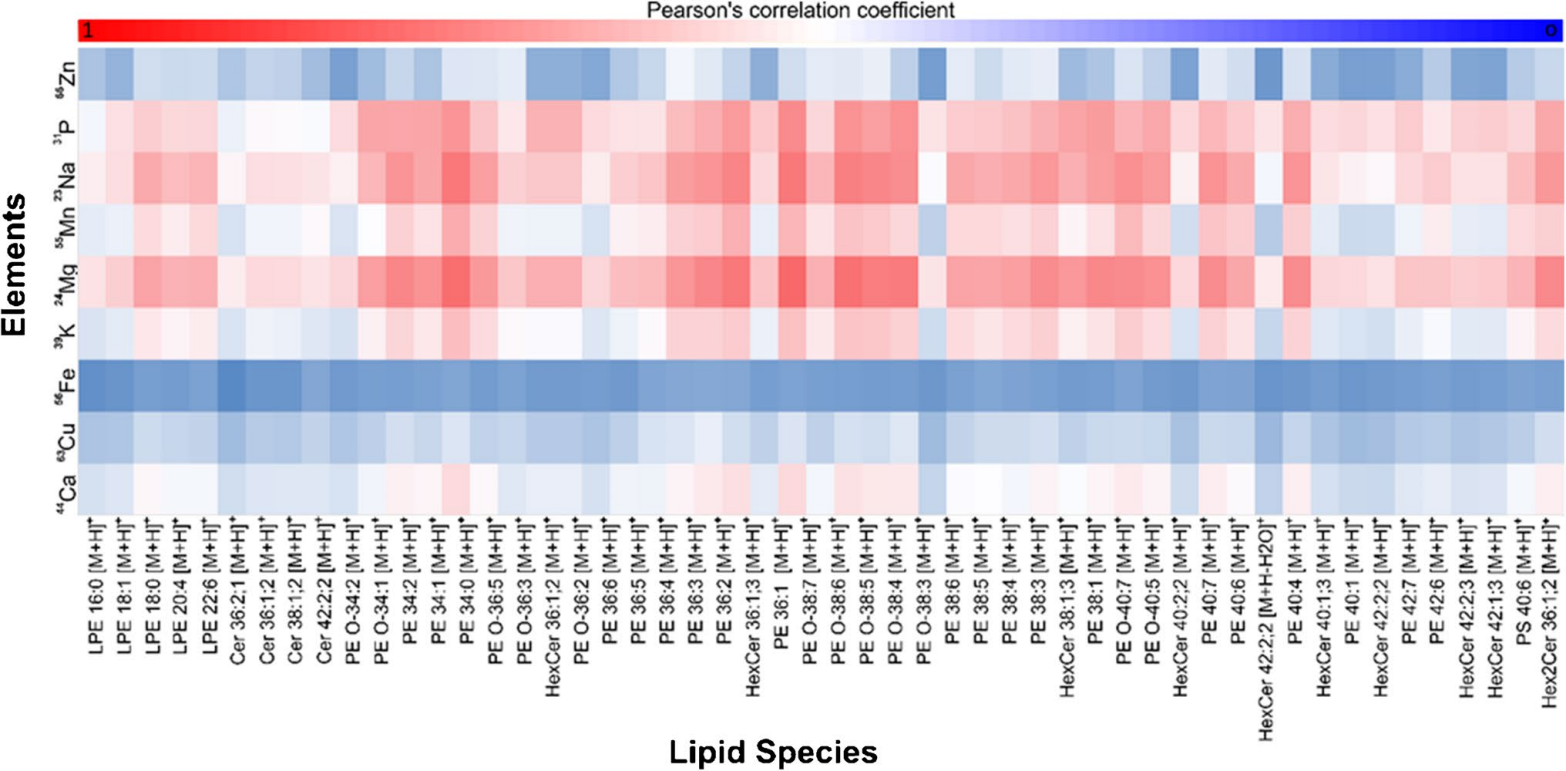
Heatmap of the spatial correlation (Pearson’s correlation) between molecular and elemental mass spectrometry imaging of workflow outlined on slide E. Hexosylceramide (HexCer) is used for glucosyl and galactosyl moieties, ceramides (Cer), phosphatidylethanolamines (PE), lysophosphatidylethanolamine (LPE), and phosphatidylserine (PS)

The heatmap in Fig. 6 shows the correlation between a variety of lipid species representing classes well-detected by AP-MALDI-PPI, including ceramides, HexCer, and PE lipids, and the elements analysed in this study: ^23^Na, ^24^ Mg, ^31^P, ^39^ K, ^44^Ca, ^55^Mn, ^56^Fe, ^63^Cu, and ^66^Zn (note red indicates high spatial correlation). A table with their respective correlation numbers can be found in Electronic Supplementary Material Table S2.

The resulting correlation heatmap shows a general correlation between lipids and the elemental images for traces of ^24^ Mg, ^55^Mn, ^23^Na, ^39^ K, and ^31^P. ^56^Fe was found to be the element with the lowest correlation for all lipid species across the board. The correlation between iron and the investigated lipid species could be explained due to the low intensity of ^56^Fe trace arising more from dorsal blood vessels and ventricles. The lipid species that presented the highest resemblance with the spatial distribution for the elements herein analysed is identified as [PE 36:1 + H]^+^/[PC 34:1-CH_3_ + 2H]^+^. The co-registration between this lipid ion at *m/z* 746.5672 and the element trace of ^24^ Mg is found to be the highest correlation across the heatmap showing a value of 0.82. [PE 36:1 + H]^+^/[PC 34:1-CH_3_ + 2H]^+^ and ^24^ Mg traces are distributed homogeneously throughout the mouse brain tissue with a highlight in the pyramidal cell layer of the hippocampus (Electronic Supplementary Material Fig. S7).

**Fig. 7:**
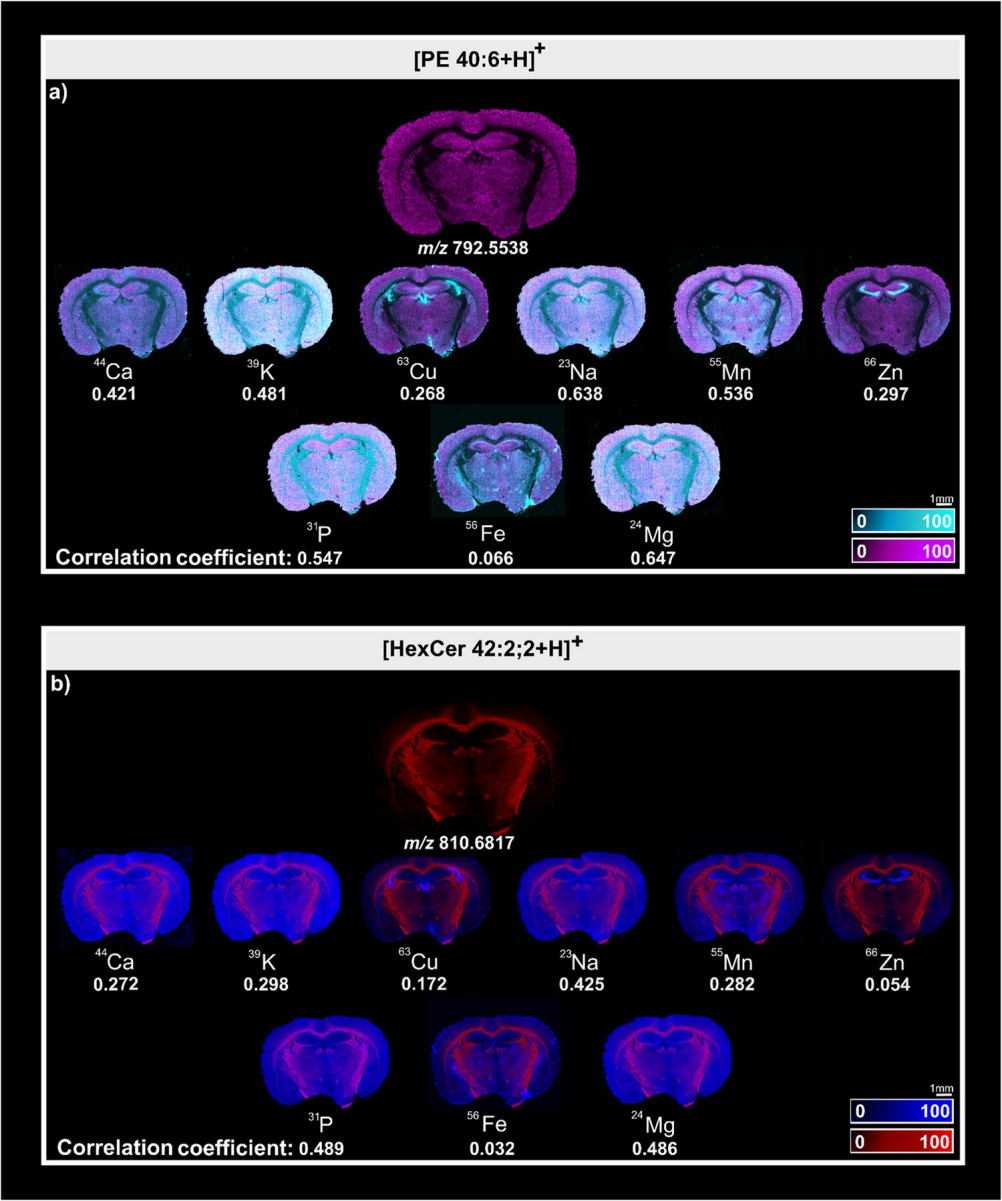
AP-MALDI-PPI and LA-ICP image co-registration. **a** Image overlay of [PE 40:6 + H]^+^ (magenta) with ^23^Na, ^24^ Mg, ^31^P, ^39^ K, ^44^Ca, ^55^Mn, ^56^Fe, ^63^Cu, and ^66^Zn (cyan) and their respective correlation coefficient. **b** Image overlay of [HexCer 42:2;2 + H]^+^ (red) with ^23^Na, ^24^ Mg, ^31^P, ^39^ K, ^44^Ca, ^55^Mn, ^56^Fe, ^63^Cu, and ^66^Zn (blue) and their respective correlation coefficient

Figure 7a and b illustrates the spatial co-registration of the ions at *m/z* 792.5538 and *m/z* 810.6817 with ^23^Na, ^24^ Mg, ^31^P, ^39^ K, ^44^Ca, ^55^Mn, ^56^Fe, ^63^Cu, and ^66^Zn. The ion at *m/z* 792.5538 assigned as [PE 40:6 + H]^+^ is the highest intensity signal and is distributed within the gray matter with more pronounced intensities arising in the cortex. The elements that show the highest correlation with the distribution observed for [PE 40:6 + H]^+^ are ^24^ Mg and ^23^Na with a correlation coefficient value of 0.647 and 0.638, respectively. Whilst ^63^Cu and ^66^Zn show the lowest correlation values after ^56^Fe (0.066). The ion distribution shown in Fig. 7b at *m/z* 810.6817, assigned as [HexCer 42:2;2 + H]^+^, is observed to arise from the myelin-rich white matter. This species presents low correlation values for all elements analysed with the highest value being 0.489 for ^31^P trace. Both molecular distributions arising from [PE 40:6 + H]^+^ and [HexCer 42:2;2 + H]^+^ are in agreement with previous reports [27].

## Conclusion

This work has further demonstrated the possibilities of combining elemental and biomolecular MSI on the same tissue section using two complementary MSI techniques (AP-MALDI-PPI for lipid imaging and LA-ICP for elemental imaging). Building off earlier studies that used MALDI and LA-ICP [44], this work is the first to combine post-ionisation technologies for lipid imaging and LA-ICP-MS from the same tissue section. It was found that analysing matrix-coated samples by LA-ICP yielded comparable spatial distributions for endogenous elements relative to the analysis of native tissue sections and that both spraying and sublimation were compatible matrix application methods to couple with LA-ICP. Moreover, removal of the matrix prior to LA-ICP was shown to still preserve the distribution of less mobile elements, whilst more mobile ones experienced delocalisation. Whilst the acquisition of AP-MALDI-PPI data prior to LA-ICP was beneficial due to the reduced tissue damage in the first analysis, this study also demonstrated that it is possible to first acquire LA-ICP-MSI data using reduced laser energies that reduce tissue ablation. However, this likely leads to some artefacts due to the reduced ablation laser energies which might lead to different ion distributions being observed and should be evaluated on a case-by-case basis. Nonetheless, subsequent AP-MALDI-PPI acquisition yielded similar lipid data than when it was performed prior to LA-ICP.

Taken together, this work expands the repertoire of multimodal MSI approaches that enable sequential lipid and elemental imaging from the same tissues. As the AP-MALDI-PPI method still uses MALDI matrices, it is also likely that the approach is applicable to other MALDI-MSI workflows and for other analyte classes.

## Supplementary Information

Below is the link to the electronic supplementary material.

Supplementary file1 (PDF 1.36 MB)

## Declarations

Animal tissues were provided as residual tissue from other animal studies at the University of Wollongong in accordance with the Australian code for the care and use of animals for scientific purposes.
