# Supplementary material for: Evaluation of combined workflows for multimodal mass spectrometry imaging of elements and lipids from the same tissue section
Source: Anal Bioanal Chem. 2025 Jan 20;417(4):705–19. doi: 10.1007/s00216-024-05696-w (PMC11772510; doi:10.1007/s00216-024-05696-w)
Supplement: Supplementary file 1 — Supplementary file1 (PDF 1.36 MB) [file 216_2024_5696_MOESM1_ESM.pdf]

Analytical and Bioanalytical Chemistry

SUPPLEMENTARY INFORMATION

Evaluation of Combined Workflows for Multimodal Mass Spectrometry Imaging of Elements and Lipids from the Same Tissue Section

Tassiani Sarretto[1], Mika T. Westerhausen[2], Jayden C. Mckinnon[1], David P. Bishop[2], Shane R. Ellis*[1]

[1]Molecular Horizons and School of Chemistry and Molecular Bioscience, University of Wollongong, Australia
[2]Hyphenated Mass Spectrometry Laboratory, University of Technology Sydney, Ultimo, Sydney, NSW, Australia.

*to whom correspondence should be addressed
Email: sellis@uow.edu.au

Table of Contents

Figure S1 3
Figure S2 3
Figure S3 4
Figure S4 5
Figure S5 6
Figure S6 6
Table S1 7
Table S2 7
Figure S7 9

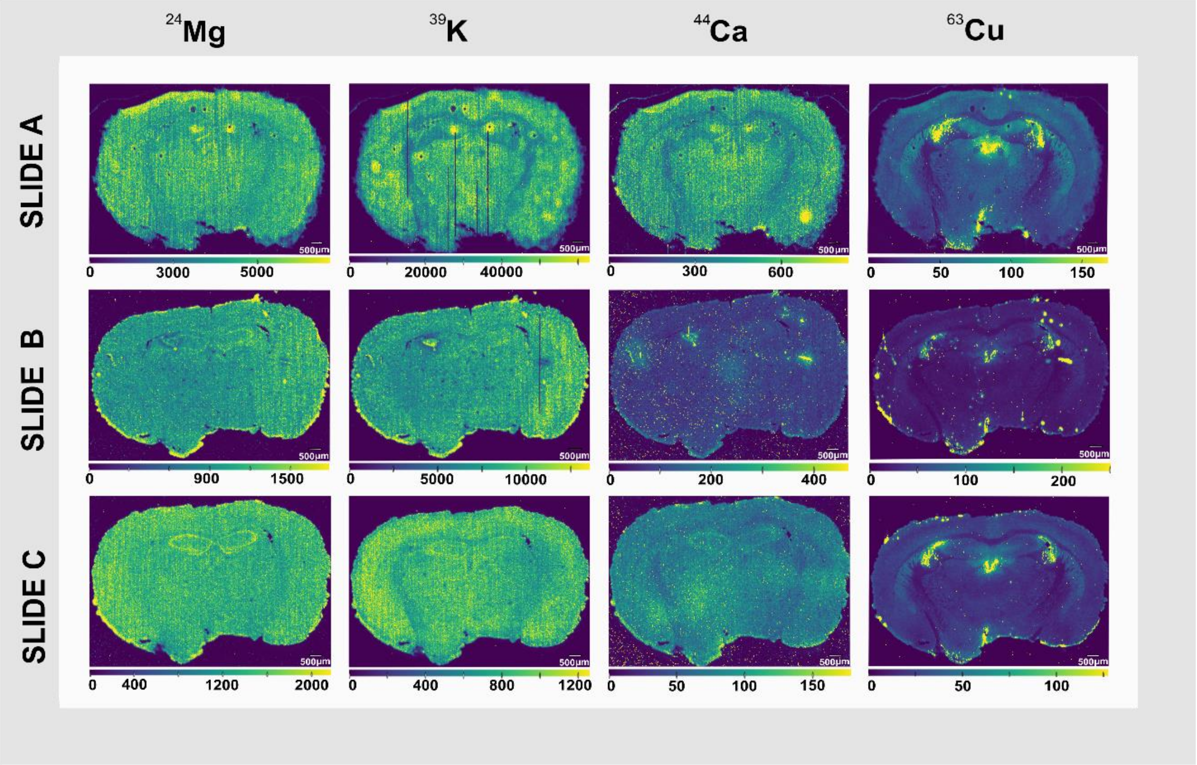

Fig. S1 Elemental images of the remaining elements not shown in Figure 2. Slide A was analysed as the native tissue without matrix deposition. Slide B was spray-coated in matrix and slide C was coated in matrix via sublimation. ^{24}Mg and ^{63}Cu are in mg/kg and ^{39}K and ^{44}Ca are in counts/second (see methods).

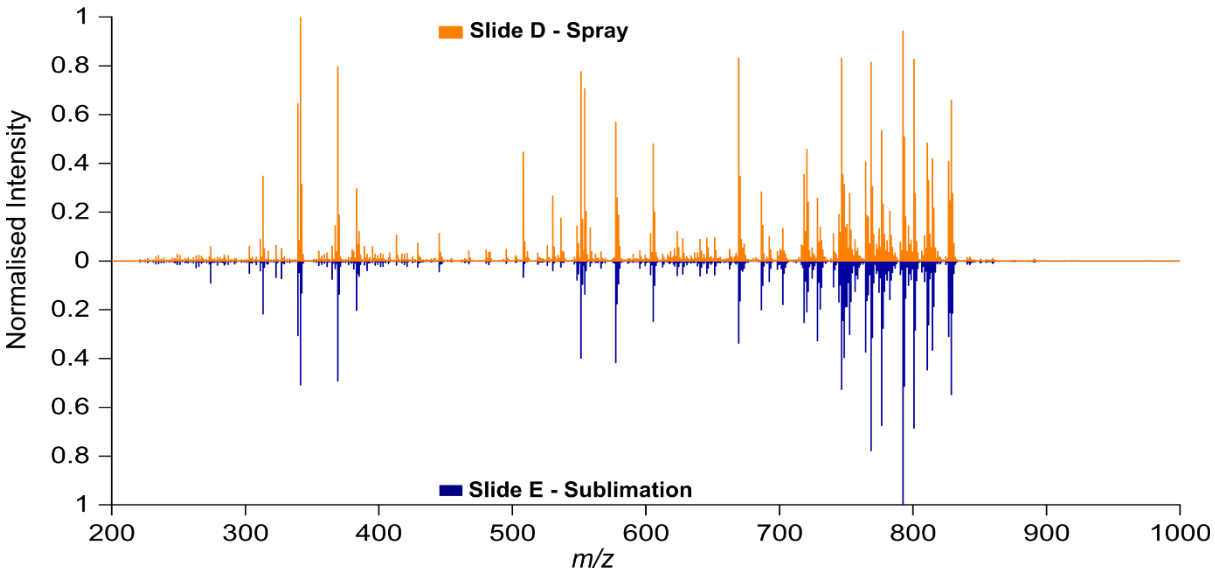

Fig. S2 Full mass range AP-MALDI-PPI mass spectra for slide D (spray-coated, orange trace) and slide E (sublimated-coated, blue trace) shown in Figure 3.

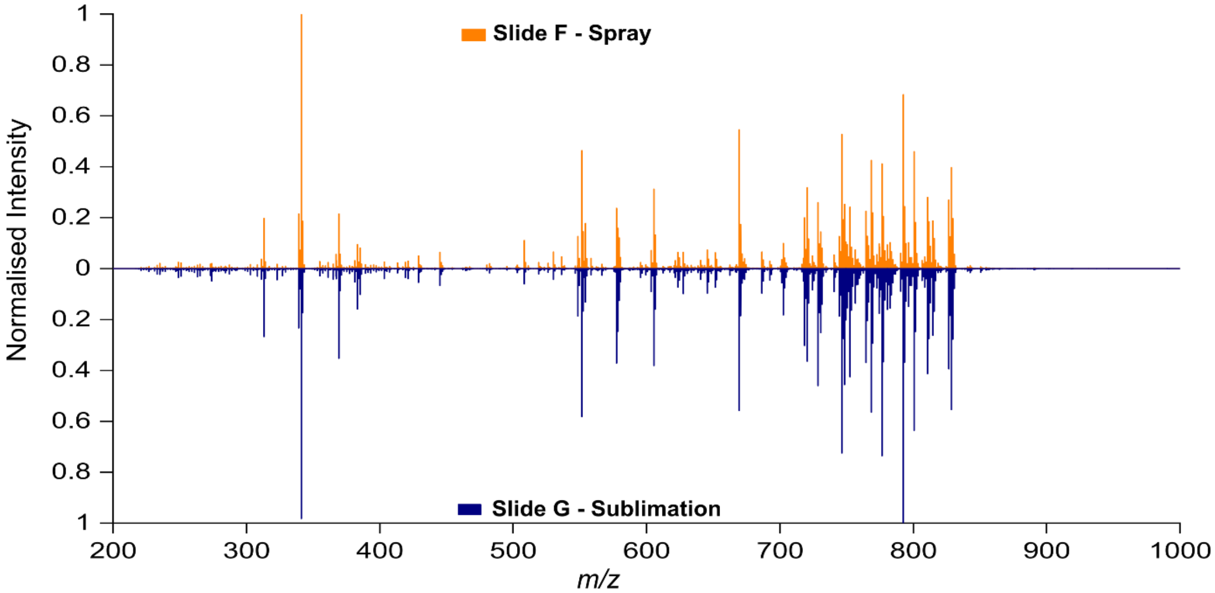

Fig. S3 Full mass range AP-MALDI-PPI mass spectra for slide F (spray-coated, orange trace) and slide G (sublimated-coated, blue trace) shown in Figure 4.

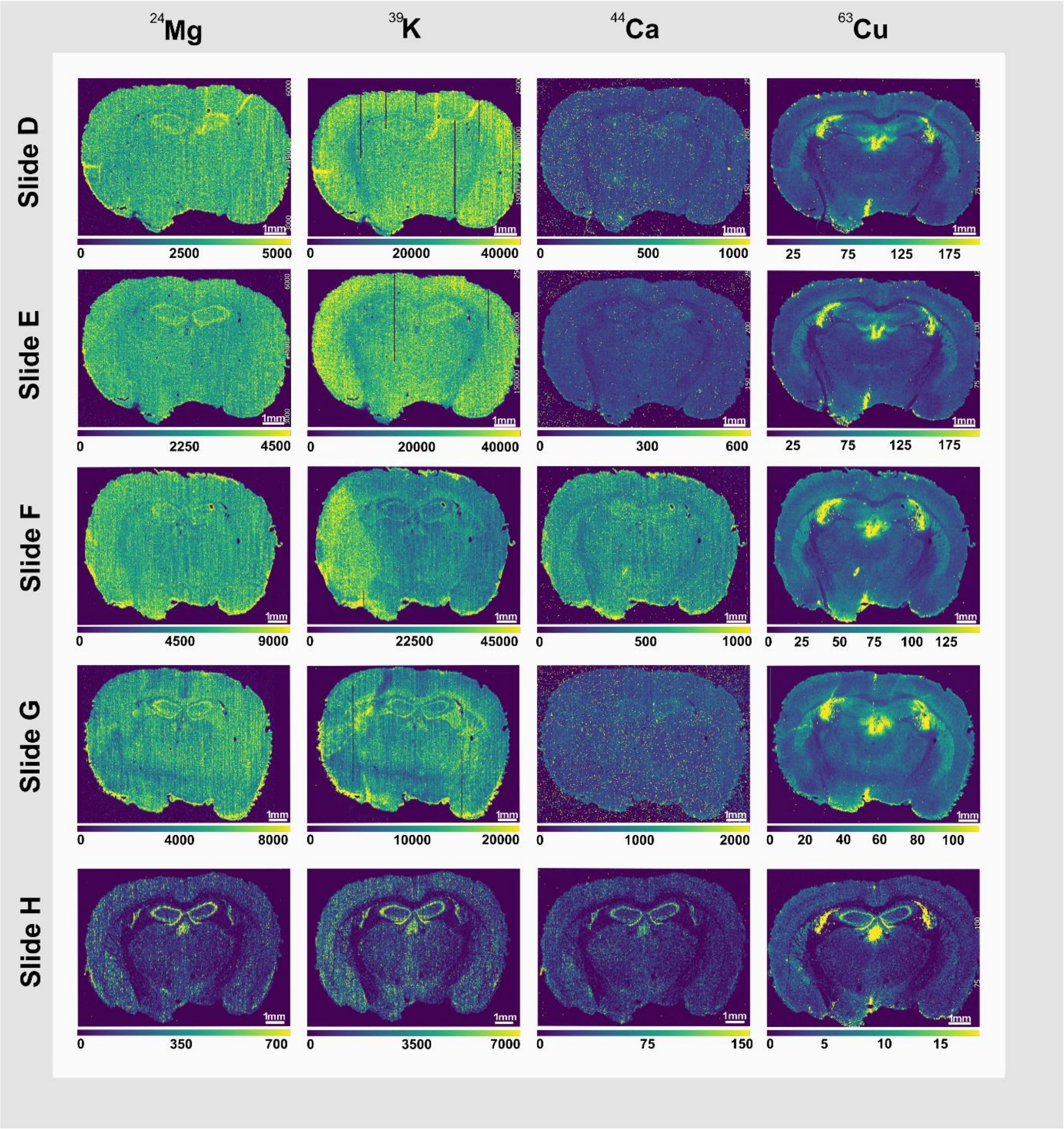

Fig. S4 LA-ICP-MSI data for elements not shown in the main text for workflows D, E, F, G and H. ^{24}Mg and ^{63}Cu are in mg/kg and ^{39}K and ^{44}Ca are in counts/second (see methods).

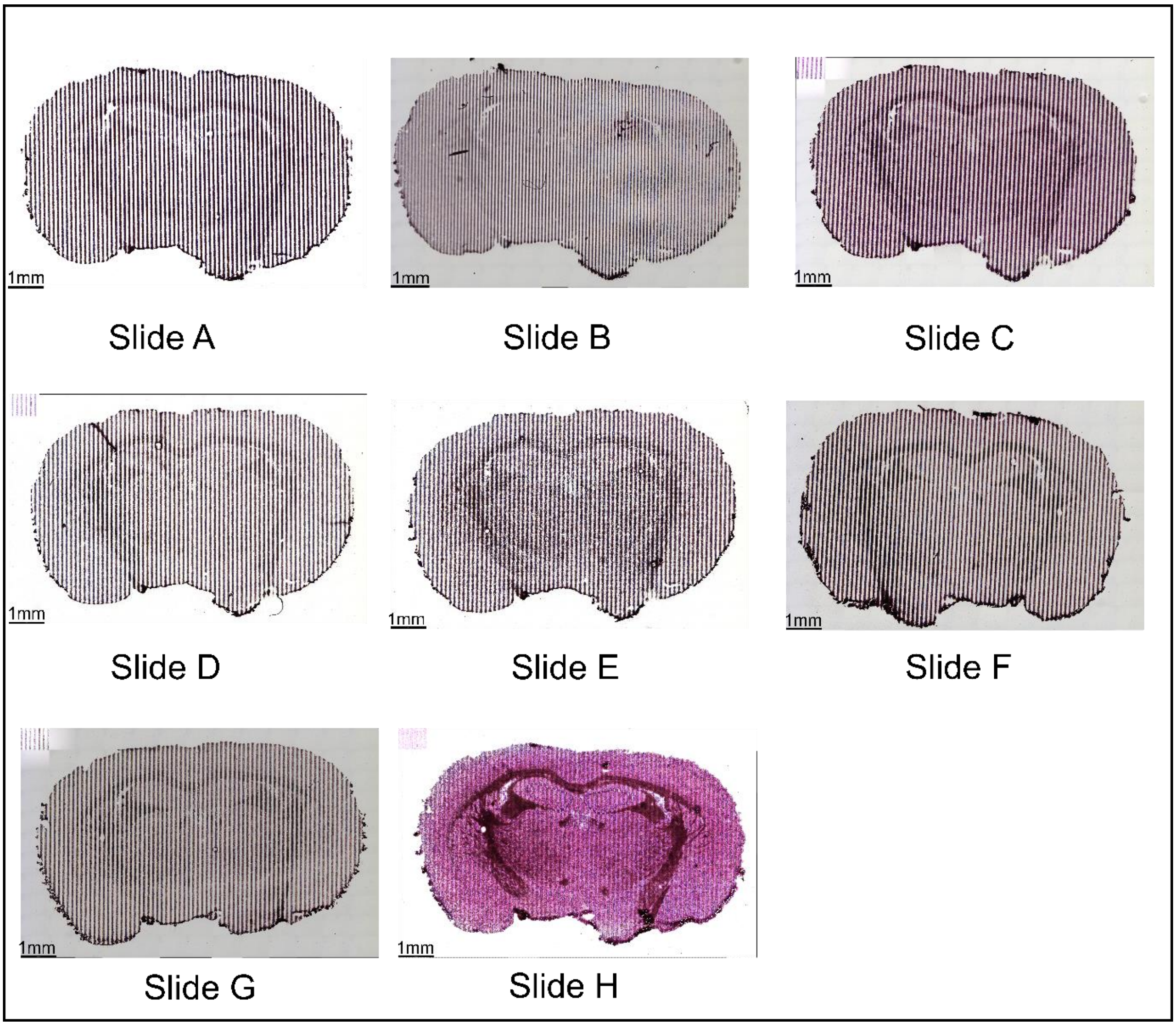

Fig. S5 Optical images of H&E-stained tissue sections used for acquisition of workflows A-H.

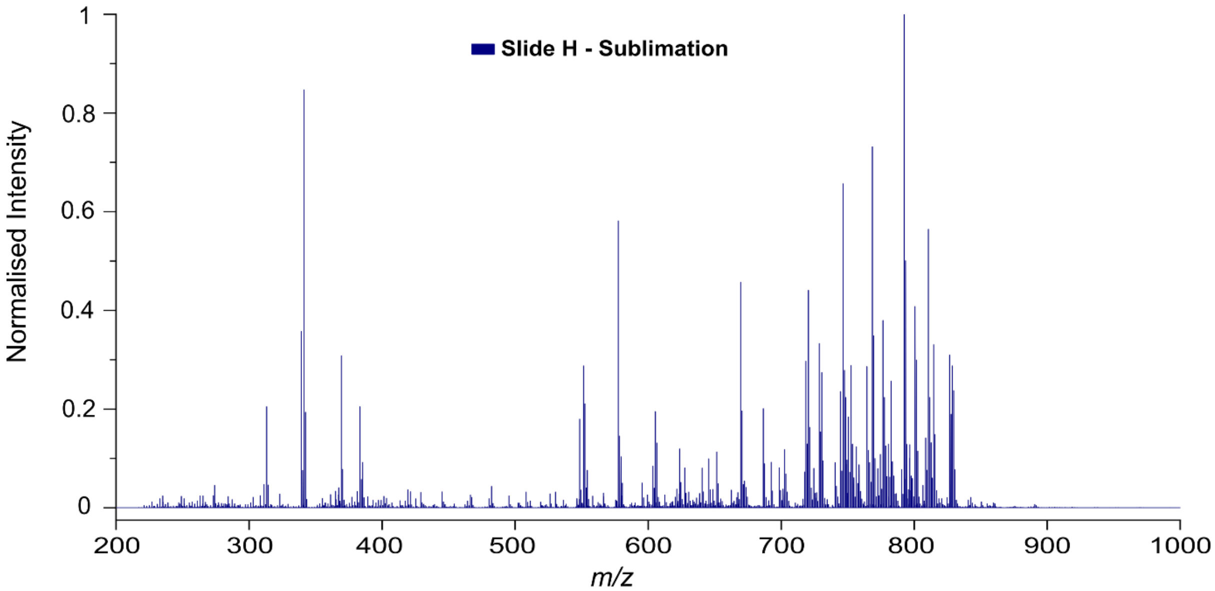

Fig. S6 Averaged mass spectra of mouse brain tissue analysed via AP-MALDI-PPI-MSI for workflow H.

Table S1 Species used for timsTOF mass calibration.

Species	Reference Mass (*m/z*)	Molecular Formula	Ion adduct
FA 18:1	283.2632	$C_{18}H_{35}O_2$	$[M+H]^+$
FA 20:4	305.2475	$C_{20}H_{33}O_2$	$[M+H]^+$
FA 22:6	329.2475	$C_{22}H_{33}O_2$	$[M+H]^+$
Cholesterol	369.3516	$C_{27}H_{45}$	$[M+H-H_2O]^+$
PE 32:0	692.5225	$C_{37}H_{75}NO_8P$	$[M+H]^+$
PE 36:1	746.5694	$C_{41}H_{81}NO_8P$	$[M+H]^+$
PE 40:6	792.5538	$C_{45}H_{79}NO_8P$	$[M+H]^+$
HexCer 42:2;2	810.6817	$C_{48}H_{92}NO_8$	$[M+H]^+$
PI 38:4	904.5910	$C_{47}H_{87}NO_{13}P$	$[M+NH_4]^+$

Table S2 Spatial correlation between lipids and elements measured by LA-ICP-MSI and AP-MALDI-PPI-MSI, respectively, from workflow E.

Measured mass (*m/z*)	ppm error	Assignment	Ca^{44}	Cu^{63}	Fe^{56}	K^{39}	Mg^{24}	Mn^{55}	Na^{23}	P^{31}	Zn^{66}
454.2948	4.4	LPE 16:0 $[M+H]^+$	0.306	0.196	-0.008	0.315	0.485	0.349	0.452	0.389	0.192
480.3074	-2.2	LPE 18:1 $[M+H]^+$	0.328	0.202	0.010	0.344	0.536	0.364	0.498	0.489	0.129
482.3241	0.0	LPE 18:0 $[M+H]^+$	0.427	0.284	0.050	0.471	0.657	0.504	0.639	0.541	0.291
502.2918	-2.0	LPE 20:4 $[M+H]^+$	0.394	0.263	0.039	0.438	0.616	0.457	0.588	0.513	0.278
526.2920	-1.6	LPE 22:6 $[M+H]^+$	0.392	0.254	0.061	0.459	0.624	0.510	0.612	0.517	0.283
546.5228	-3.0	Cer 36:2;1 $[M+H]^+$	0.291	0.163	-0.034	0.315	0.456	0.310	0.434	0.368	0.202
548.5377	-4.4	Cer 36:1;2 $[M+H]^+$	0.327	0.220	0.014	0.375	0.509	0.381	0.494	0.422	0.255
566.5504	-0.5	Cer 36:1;2 $[M+H]^+$	0.330	0.212	0.013	0.364	0.505	0.382	0.492	0.416	0.246
576.5696	-3.1	Cer 38:1;2 $[M+H]^+$	0.330	0.252	0.079	0.331	0.481	0.424	0.478	0.405	0.167
648.6290	0.1	Cer 42:2;2 $[M+H]^+$	0.310	0.200	0.032	0.308	0.513	0.318	0.448	0.500	0.054
702.5424	-1.2	PE O-34:2 $[M+H]^+$	0.382	0.240	0.048	0.445	0.670	0.413	0.603	0.650	0.163
704.5581	-1.1	PE O-34:1 $[M+H]^+$	0.448	0.298	0.046	0.519	0.739	0.531	0.698	0.649	0.266
716.5249	3.4	PE 34:2 $[M+H]^+$	0.434	0.270	0.057	0.469	0.703	0.484	0.647	0.650	0.194
718.5359	-3.1	PE 34:1 $[M+H]^+$	0.507	0.322	0.073	0.582	0.805	0.633	0.777	0.696	0.327
720.5506	-4.4	PE 34:0 $[M+H]^+$	0.425	0.251	0.040	0.503	0.686	0.526	0.668	0.560	0.331
724.5282	0.9	PE O-36:5 $[M+H]^+$	0.334	0.264	0.064	0.423	0.559	0.379	0.533	0.470	0.350
728.5569	-2.7	PE O-36:3 $[M+H]^+$	0.357	0.219	0.035	0.403	0.624	0.374	0.558	0.617	0.111
728.6006	-4.0	HexCer 36:1;2 $[M+H]^+$	0.357	0.219	0.035	0.403	0.624	0.374	0.558	0.617	0.111
730.5741	-0.6	PE O-36:2 $[M+H]^+$	0.308	0.195	0.043	0.327	0.516	0.310	0.457	0.505	0.084
736.4900	-1.6	PE 36:6 $[M+H]^+$	0.378	0.239	0.027	0.374	0.582	0.442	0.538	0.483	0.209
738.5071	0.4	PE 36:5 $[M+H]^+$	0.372	0.310	0.070	0.420	0.592	0.456	0.563	0.481	0.266

740.5222	-0.4	PE 36:4 $[M+H]^+$	0.438	0.323	0.081	0.525	0.694	0.530	0.675	0.586	0.384
742.5355	-3.5	PE 36:3 $[M+H]^+$	0.464	0.358	0.089	0.525	0.734	0.544	0.697	0.628	0.337
744.5542	0.6	PE 36:2 $[M+H]^+$	0.491	0.294	0.082	0.547	0.777	0.621	0.741	0.681	0.256
744.5952	-4.3	HexCer 36:1;3 $[M+H]^+$	0.346	0.245	0.045	0.365	0.565	0.356	0.512	0.550	0.109
746.5672	-3.0	PE 36:1 $[M+H]^+$	0.509	0.320	0.062	0.576	0.817	0.605	0.776	0.725	0.290
748.5258	-2.4	PE O-38:7 $[M+H]^+$	0.388	0.246	0.048	0.468	0.622	0.482	0.605	0.514	0.345
750.5420	-1.6	PE O-38:6 $[M+H]^+$	0.488	0.306	0.045	0.567	0.795	0.569	0.755	0.702	0.339
752.5562	-3.5	PE O-38:5 $[M+H]^+$	0.468	0.290	0.039	0.560	0.763	0.553	0.736	0.666	0.361
754.5720	-3.3	PE O-38:4 $[M+H]^+$	0.467	0.330	0.043	0.532	0.770	0.514	0.717	0.707	0.240
756.5894	-1.0	PE O-38:3 $[M+H]^+$	0.259	0.158	0.023	0.286	0.478	0.244	0.410	0.482	0.044
764.5219	-0.8	PE 38:6 $[M+H]^+$	0.410	0.253	0.058	0.504	0.657	0.510	0.647	0.545	0.345
766.5352	-3.8	PE 38:5 $[M+H]^+$	0.410	0.285	0.080	0.474	0.651	0.510	0.627	0.550	0.283
768.5538	0.0	PE 38:4 $[M+H]^+$	0.389	0.286	0.067	0.506	0.669	0.491	0.645	0.572	0.341
770.5662	-4.2	PE 38:3 $[M+H]^+$	0.436	0.300	0.052	0.533	0.720	0.528	0.687	0.618	0.353
772.6284	-1.7	HexCer 38:1;3 $[M+H]^+$	0.407	0.244	0.036	0.448	0.687	0.437	0.624	0.655	0.157
774.5991	-2.1	PE 38:1 $[M+H]^+$	0.435	0.274	0.040	0.482	0.733	0.486	0.672	0.677	0.199
776.5574	-1.9	PE O-40:7 $[M+H]^+$	0.460	0.280	0.069	0.541	0.724	0.594	0.710	0.608	0.320
780.5904	0.3	PE O-40:5 $[M+H]^+$	0.436	0.270	0.036	0.500	0.707	0.513	0.680	0.643	0.246
782.6500	-0.6	HexCer 40:2;2 $[M+H]^+$	0.290	0.183	0.026	0.313	0.511	0.296	0.446	0.511	0.063
790.5355	-3.3	PE 40:7 $[M+H]^+$	0.454	0.276	0.068	0.530	0.721	0.566	0.703	0.602	0.344
792.5540	0.3	PE 40:6 $[M+H]^+$	0.421	0.268	0.066	0.481	0.647	0.536	0.638	0.547	0.297
792.6706	-0.7	HexCer 42:2;2 $[M+H-H_2O]^+$	0.249	0.148	0.008	0.266	0.463	0.222	0.391	0.480	0.023
796.5824	-3.4	PE 40:4 $[M+H]^+$	0.449	0.259	0.023	0.524	0.720	0.535	0.699	0.627	0.313
800.6593	-2.1	HexCer 40:1;3 $[M+H]^+$	0.304	0.195	0.042	0.335	0.515	0.347	0.475	0.499	0.095
802.6329	1.1	PE 40:1 $[M+H]^+$	0.277	0.159	0.017	0.314	0.508	0.281	0.443	0.515	0.052
810.6817	-0.1	HexCer 42:2;2 $[M+H]^+$	0.272	0.172	0.032	0.298	0.486	0.282	0.425	0.489	0.054
818.5672	-2.7	PE 42:7 $[M+H]^+$	0.335	0.213	0.043	0.364	0.565	0.377	0.511	0.545	0.100
820.5834	-2.0	PE 42:6 $[M+H]^+$	0.354	0.224	0.055	0.399	0.562	0.457	0.544	0.471	0.223
826.6741	-3.1	HexCer 42:2;3 $[M+H]^+$	0.317	0.196	0.040	0.341	0.540	0.354	0.485	0.531	0.078
828.6895	-3.4	HexCer 42:1;3 $[M+H]^+$	0.323	0.206	0.045	0.343	0.549	0.346	0.487	0.541	0.069
836.5452	1.9	PS 40:6 $[M+H]^+$	0.394	0.234	0.067	0.436	0.608	0.506	0.584	0.514	0.221
890.6535	-3.2	Hex2Cer 36:1;2 $[M+H]^+$	0.450	0.297	0.054	0.504	0.729	0.535	0.689	0.645	0.271

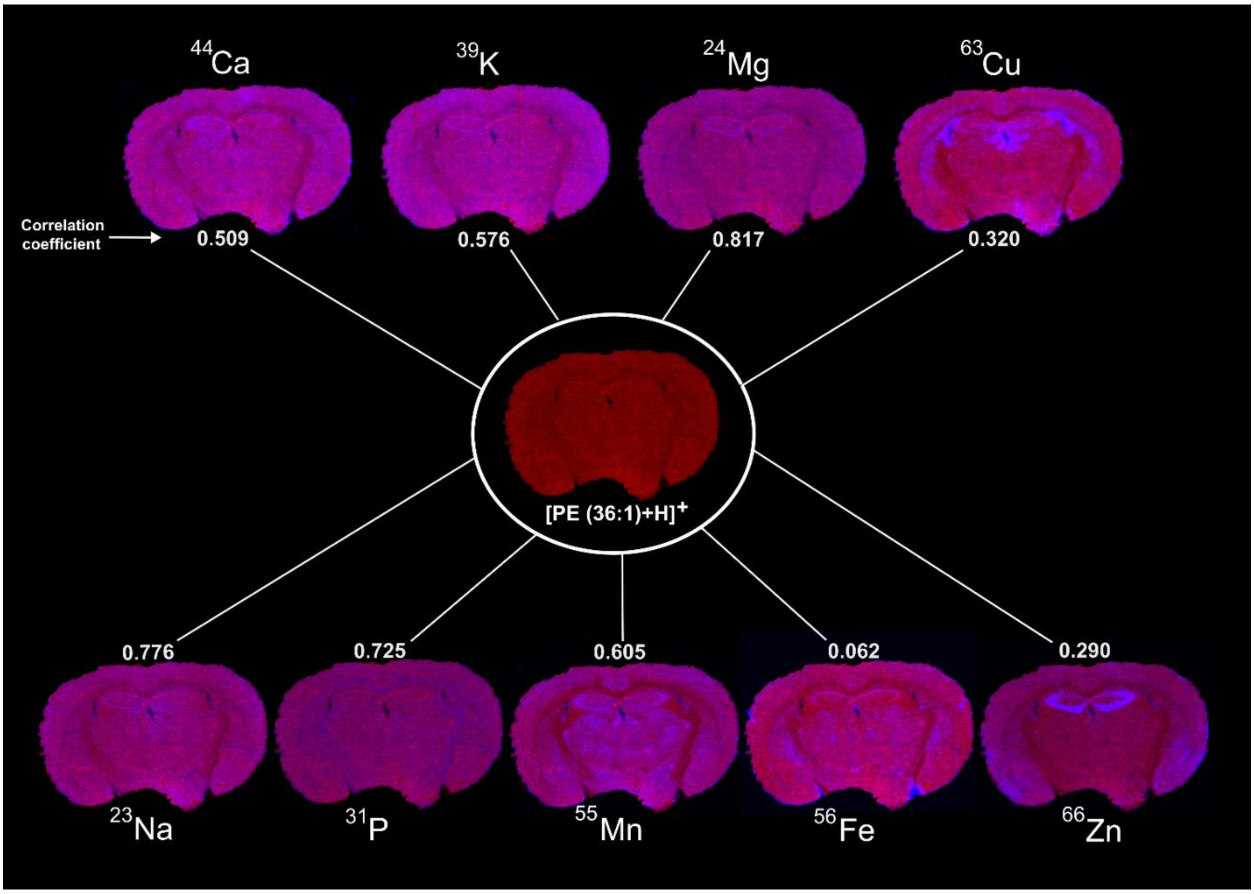

Fig. S7 Overlay of $[PE(36:1)+H]^+/[PC(34:1)-CH_3+H]^+$ measured by AP-MALDI-PPI-MSI (red) with ^{23}Na, ^{24}Mg, ^{31}P, ^{39}K, ^{44}Ca, ^{55}Mn, ^{56}Fe, ^{63}Cu and ^{66}Zn represented by blue in each image. Spatial correlation coefficients for each AP-MALDI-PPI-MSI and LA-ICP-MSI pair are also shown.
